# Postpartum Acquired Hemophilia: A Rare Cause of Postpartum Hemorrhage

**DOI:** 10.1155/2013/735715

**Published:** 2013-02-28

**Authors:** Srikanth Seethala, Sumit Gaur, Elizabeth Enderton, Javier Corral

**Affiliations:** ^1^Department of Internal Medicine, University of New Mexico-Albuquerque, 1 University of New Mexico, MSC10 555, Albuquerque, NM 87131, USA; ^2^Department of Internal Medicine, Texas Tech University Health Sciences Center, 4800 Alberta Avenue, El Paso, TX 79905, USA; ^3^Department of Obstetrics and Gynecology, Texas Tech University Health Sciences Center, 4800 Alberta Avenue, El Paso, TX 79905, USA

## Abstract

A 36-year-old female started having postpartum vaginal bleeding after normal vaginal delivery. She underwent hysterectomy for persistent bleeding and was referred to our institution. An elevation of PTT and normal PT made us suspect postpartum acquired hemophilia (PAH), and it was confirmed by low factor VIII activity levels and an elevated factor VIII inhibitor. Hemostasis was achieved with recombinant factor VII concentrates and desmopressin, and factor eradication was achieved with cytoxan, methylprednisolone, and plasmapheresis.

## 1. Introduction

Postpartum acquired hemophilia (PAH) is a rare bleeding disorder secondary to spontaneous development of inhibitors against factor VIII during peripartum period. 

## 2. Case Presentation

A 36-year-old female without any medical problems presented to our institution for normal vaginal delivery. On the day of admission, she had hemoglobin (Hb) of 11 g/dl and a hematocrit (Hct) of 35%. Her puerperal course was complicated by excessive vaginal bleeding and a drop in the hemoglobin from 11 to 6.8 g/dl. She refused blood transfusions. She returned to Mexico and continued to have bleeding and sought medical attention in Mexico. Chorioamnionitis was diagnosed by hysteroscopy and received intravenous antibiotics. Hysterectomy was performed for persistent vaginal bleeding that was complicated by intraabdominal hemorrhage, intraabdominal abscess, and two subsequent wash outs. Her condition continued to deteriorate and was referred back to our institution with a diagnosis of disseminated intravascular coagulation (DIC).

She was intubated for hemodynamic instability. Her initial lab work revealed white blood cell count of 19.4/microliter, Hb 6.9 g/dl, Hct 19.5%, platelet count of 136/microliter, PTT of 71.7 sec, PT of 15.9 sec, international normalized ratio (INR) 1.3, fibrinogen 369 mg/dl (234–500), and fibrin degradation products (FDP) greater than 5 micrograms/ml (<20 normal). DIC was considered. CT scan of the abdomen revealed probable retained sponges and exploratory laparotomy was performed after transfusing packed red blood cells (PRBCs), fresh frozen plasma (FFP), and cryoprecipitate. Hemostasis was initially achieved with FFP. However, postoperatively, intraabdominal hemorrhage reoccurred and could not be controlled with multiple FFP and cryoprecipitate transfusions.

Medical services were consulted for coagulopathy evaluation. PAH was suspected because of isolated elevation of PTT, normal PT, INR, fibrinogen, and FDP. Mixing studies, factor assays, and inhibitor levels were performed. Hemostasis was achieved with recombinant factor VII concentrates and desmopressin. The factor VIII activity level was less than 1 with a factor VIII inhibitor at a concentration of 54.3 Bethesda units (very high), thus confirming the diagnosis of PAH. Methylprednisolone, Cytoxan, and plasmapheresis were prescribed. The patient responded with an appropriate decline in PTT, but her hospital course was complicated by the vesico-vaginal fistula, recto-vaginal fistula, and C diff colitis and bacteremia. We discontinued the immunosuppressive therapy and discharged after inhibitor levels were undetectable. Unfortunately, one month later she was readmitted to the hospital with severe sepsis and subsequently died.

## 3. Discussion

Postpartum acquired hemophilia (PAH) is a rare bleeding disorder that commonly occurs one to four months after the delivery. There are case reports of PAH from as early as the antepartum to as late as 1-year postpartum [1–5]. 

It commonly presents as severe ecchymosis, soft tissue hematomas, hemarthrosis, and severe life-threatening hemorrhage [3–5]. Vaginal bleeding is the presenting symptom if the inhibitor appears early in the course, while ecchymosis predominate if it appears late. There are a few case reports about intraplacental transfer of IgG antibodies and intracerebral hemorrhage in neonates [6].

PAH should be considered as an etiology in postpartum patients with isolated elevation in PTT. An abnormal mixing study, low factor VIII levels and high inhibitor levels, confirms the diagnosis. Levels of inhibitor may correlate with the severity of the bleeding and clinical presentation.

Management of PAH has two steps. First, is to achieve hemostasis, and second is inhibitor eradication. The agent of choice for hemostasis depends on bleeding severity and inhibitor titers. FEIBA (factor VIII inhibitor bypassing activity) or recombinant factor VIIa are agents of choice in life-threatening bleeding conditions, while human factor VIII concentrates are used during non-life-threatening cases. Desmopressin can be used during minor bleeding episodes [1–7]. There are no consensuses on factor eradication treatment. Even without immunosuppressive therapy, 100% complete remission was reported. Immunosuppressive therapy decreases the course duration. Response rate neither correlates with baseline inhibitor levels nor with severity of the presentation [2, 4]. Immunosuppressive therapy with steroids either with or without cytotoxic drugs is the treatment of choice; however, the benefits must outweigh the risks, and this might vary on a case per case basis. Relapses are not uncommon and there is no correlation either with inhibitor titers or response time. Remission during subsequent pregnancies is rare but has been reported. Careful followup is needed as the inhibitor can cross the placenta [2].
